# Automatic Classification of Particles in the Urine Sediment Test with the Developed Artificial Intelligence-Based Hybrid Model

**DOI:** 10.3390/diagnostics13071299

**Published:** 2023-03-30

**Authors:** Muhammed Yildirim, Harun Bingol, Emine Cengil, Serpil Aslan, Muhammet Baykara

**Affiliations:** 1 Department of Computer Engineering, Malatya Turgut Ozal University, Malatya 44200, Turkey; 2 Department of Software Engineering, Malatya Turgut Ozal University, Malatya 44200, Turkey; 3 Department of Computer Engineering, Bitlis Eren University, Bitlis 13100, Turkey; 4 Department of Software Engineering, Firat University, Elazig 23100, Turkey; mbaykara@firat.edu.tr

**Keywords:** classification, CNN, kidney, mRMR, urine sediment

## Abstract

Urine sediment examination is one of the main tests used in the diagnosis of many diseases. Thanks to this test, many diseases can be detected in advance. Examining the results of this test is an intensive and time-consuming process. Therefore, it is very important to automatically interpret the urine sediment test results using computer-aided systems. In this study, a data set consisting of eight classes was used. The data set used in the study consists of 8509 particle images obtained by examining the particles in the urine sediment. A hybrid model based on textural and Convolutional Neural Networks (CNN) was developed to classify the images in the related data set. The features obtained using textural-based methods and the features obtained from CNN-based architectures were combined after optimizing using the Minimum Redundancy Maximum Relevance (mRMR) method. In this way, we aimed to extract different features of the same image. This increased the performance of the proposed model. The CNN-based ResNet50 architecture and textural-based Local Binary Pattern (LBP) method were used for feature extraction. Finally, the optimized and combined feature map was classified at different machine learning classifiers. In order to compare the performance of the model proposed in the study, results were also obtained from different CNN architectures. A high accuracy value of 96.0% was obtained in the proposed model.

## 1. Introduction

Every year, 830,000 people die worldwide due to kidney and urinary tract diseases [1]. It is a known fact that urine samples are taken from patients to be used in the diagnosis of many diseases, especially diabetes, metabolic, urinary, and kidney diseases. Urine culture gives information about the presence of infection in the urine. If bacteria are found in human urine, which is sterile under normal conditions, urinary tract infections develop, and antibiotics are usually used to treat this disease. In addition, the amount of excess protein or sugar in the urine contains essential information about many kidney diseases, especially kidney failure [2]. After the urine samples are taken from the patient, biochemists analyze these urine samples in detail.

Many types of particles can be found in these urine samples. While analyzing the urine sediment, particles such as erythrocyte, cylinders, leukocyte, crystals, epithelium, bacteria, yeast, and sperm can be observed. In traditional methods, biochemists classify the classes in which these particles are found by using manual methods. The process of classifying the particles in the urine is a very complex and difficult process, especially in the presence of a large number of images. In addition, problems are frequently encountered in distinguishing particles of different classes that are similar to each other in manual examinations [3]. The more accurately the urine sediment particles can be classified, the easier it is for the specialist to diagnose the patient’s disease. The earlier the disease can be diagnosed by specialists, the more likely it is that treatment can be started and result in a successful treatment. Considering both the workload caused by manual classification processes and the incorrect classification that may occur during these processes, the necessity of using computer-aided methods in particle classification processes has emerged. It is recognized by the scientific world that artificial intelligence techniques are often used among computer-assisted methods when classifying objects. Among artificial intelligence methods, deep learning techniques have become very popular in recent years due to their high success in diagnosing various diseases [4,5].

The presence of these particles in the urine is extremely important for the diagnosis of the disease for the specialist who requests the urine sediment analysis from the patient. In addition, the presence of several urine particles together in the same urine sample means that different diseases may occur.

### 1.1. Related Researches

It is very important to classify the particles in the urine using artificial intelligence-based methods. Therefore, researchers have carried out studies on the subject. Some of the studies in the literature that automatically classify the particles in the urine using deep learning methods are as follows:

Suhail et al. presented a comprehensive review study to compare the performance of artificial intelligence techniques that classify microscope images of urine particles [1].

Li et al. in their study used a data set containing 15,360 images with 16 classes consisting of urine particles. It was stated that 70 percent of the images in the data set were used for training and the rest for testing. They developed a ResNet-based method called RetinaNet. It was stated that an 88.65% accuracy value was obtained with this developed method [6].

Liang et al. stated that they used Faster R-CNN and a single-shot multi-box detector (SSD) together for the detection and classification of urinary particles. They also proposed a scheme called Trimmed SSD, which is based on removing several convolutional layers that will increase the performance of the SSD. They stated that a data set containing 7 classes and 5376 images was used during the experiments and the average precision was 84.1% [7].

Nagai et al. used a data set of 441 images and 15 classes to classify urinary sediment crystals. They increased the number of images to 60,000 by multiplying the data set they created by collecting images from different sources. They stated that the duplication processes had positive effects on the classification accuracy percentage. They stated that they obtained results with the Xception architecture from CNN architectures and stated that they obtained an accuracy value of 84.40% [8].

Chen et al. stated in their study that segmentation processes are difficult due to the low contrast values of urine sediment images. To overcome this problem, they proposed the YOLOv5s-CBL method, which treats urine sediment particle images as an object. It was stated that promising results were obtained in this study, in which two different data sets were used [9].

Khalid et al. stated that they created a 4-class data set containing 820 urine sediment images. They stated that they used MobileNet, VGG16, DenseNet, ResNet, and InceptionV3 architectures to classify urine sediment images. It was stated that DenseNet and InceptionV3 architectures achieved a 96.5% accuracy value [10].

Lee et al. stated in their study that the bacteria in the urine will show different Gram-staining reactions, so the images of these bacteria will be different. Urine images created by artificially produced bacteria were used for training in the DenseNet deep model and it was stated that the model was tested on urine particle images taken from real patients. It was stated that 263 test images were used during the experiments. It was reported that the proposed method reaches 90.9% accuracy even at low bacterial density [11].

Ji et al. proposed a semi-supervised network model to classify urine sediment images in their study. In particular, they aimed to classify low-resolution urine sediment images with high performance. During the experiments, a data set containing 429,605 urine sediment images with 16 classes was used. They stated that they obtained a 94% accuracy value with the model called US-RepNet that they suggested [12].

Li et al. tried to classify urine sediment images using LeNet-5 in their study. They stated that, since the LeNet-5 manuscript was designed to recognize figures, they made some changes to this architecture in order to be able to recognize urine sediment images. At the beginning of these, they reduced the output layer from 10 to 4. They increased the number of cores in some layers and used the ReLu function instead of the sigmoid used as the activation function, thus they stated that they obtained a modified LeNet-5 architecture. During the experiments, 2551 urine sediment images consisting of 4 classes were used. They stated that they performed classification with 92% accuracy during the experiments [13].

In their study, Zhang et al. proposed a new model using the Adam optimization method to classify urine sediment particles. They stated that they increased the speed of convergence to the most suitable solution with the model they proposed [14].

### 1.2. Contributions and Novelty

The contributions and novelty of the study to the literature can be summarized as follows:

- It is different from other studies in the literature because, due to using artificial intelligence techniques in the classification of urine particles, a hybrid model is proposed, which was applied for the first time in this study and produced effective results.
- In the proposed hybrid model, the feature maps obtained separately with the ResNet50 architecture, which is one of the CNN architectures, and the LBP method are combined. In this way, different features of the images were extracted with two different methods, and then these different features were combined.
- The mRMR approach eliminates unneeded features to speed up and improve the performance of the proposed model.
- The feature map optimized by the mRMR method was classified in different machine learning classifiers.
- It was demonstrated that tissue- and CNN-based architectures work effectively together in the classification of urine particles.
- It was determined that SVM, which is one of the traditional machine learning classifiers, performs better than other traditional classifiers and Softmax in the classification of urine particles.

### 1.3. Organization of Article

The remainder of the work is organized as follows. Detailed information about the data set used in the study, the LBP method, the mRMR method, CNN architectures, and the proposed model are provided in Section 2. Experimental results are provided in Section 3. The discussion is explored in Section 4. Section 5 contains the conclusion of the study.

## 2. Material and Methods

In this section, the data set used in the study, the LBP method, mRMR, pre-trained models, classifiers, and the proposed model are examined.

### 2.1. Urine Sediment Data Set

The data set used in the experiments was obtained by the Biochemistry Clinic of Elazig Fethi Sekin City Hospital, using the Optika B293PLi microscope, with a total of 8509 urine sediment particle images from 409 patients. The urine sediment particle data set was publicly accessed from “https://github.com/ttuncer/urinedataset (24 February 2023)”and used in experiments [15]. The relevant data set is an updated data set shared in 2023. In Table 1, the number of images for each class in the data set and the total number of images are given.

**Figure 1:**
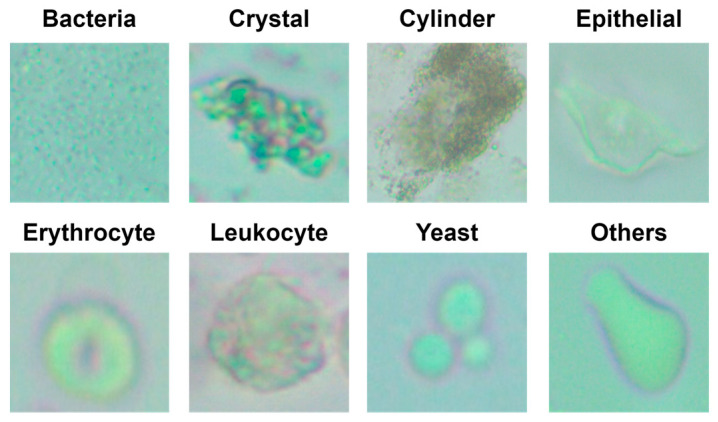
Example images of classes in the data set.

In the “Others” class, there are images outside of the seven classes presented in the study. Example images of the classes in the data set are given in Figure 1.

### 2.2. Local Binary Pattern (LBP) and Minimum Redundancy Maximum Relevance (mRMR)

**Figure 2:**
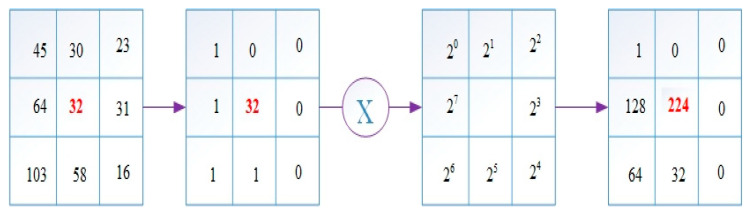
LBP operator working steps.

In the study, the Local Binary Pattern (LBP) method and CNN-based architectures were used for feature extraction. LBP, introduced by Ojala et al., is frequently used in applications based on image processing [16]. The Local Binary Pattern is based on the neighborhoods of each pixel. It is very easy to use and inexpensive. Figure 2 shows how the LBP operator works.

First, the difference between the pixel value at the selected center point and all neighboring values are checked. If the neighboring pixel value is less than the center pixel value, 0 is given as the new value, and 1 if it is larger. Then, the weight matrix is subjected to the convolution process with the obtained values. The value obtained as a result of the operation is the new value of the center point. Of the values created in this way, those with 0-1 and 1-0 transitions of 2 or less are used in the binary LBP code [17].

The features selected using the LBP method and CNN-based architectures were optimized by the mRMR method. Feature selection algorithms enable machine learning methods to run faster and achieve better results, such as reducing input size and eliminating irrelevant, residual data. The importance of feature algorithms becomes more evident, especially in data sets where there are many features with high correlation and the number of samples is small. mRMR [18] is an entropy-based feature selection method. mRMR tends to select features that are highly correlated with class and have a low correlation with each other. It is preferred because of its high-performance rate and fast operation. The mRMR algorithm has two selection requirements, referred to as mutual information difference (MID) and mutual information division (MIQ). In addition, for continuous features, two alternatives are proposed, the F-test correlation difference (FCD) and the F-test correlation coefficient (FCQ), since probability density is assumed to be computationally expensive [19].

### 2.3. CNN Architectures

Unlike conventional image classification techniques, deep learning methods do not require feature extraction or preprocessing. Instead, deep learning models carry out these tasks independently without assistance. CNN, which has a layered structure, is used for these tasks. The input layer is the first layer in CNN, followed by the convolution layer, where the image features are extracted, and the classification layer is the final layer. Convolution, pooling, activation, dropout, and FC layers are all part of the intermediate layer. This study extracts the FC layer’s features using six different CNN architectures for classification. AlexNet [20], ResNet50 [21], GoogleNet [22], EfficientNetb0 [23], MobileNetV2 [24], and ShuffleNet [25] are the top six networks.

AlexNet: AlexNet is a deep learning model created to classify images. A 227 by 227 image is fed into the input layer. The 227 MB model has 25 layers, including five convolutional, maximum-pooling, three fully-connected, 1000-way softmax, and output layers. There are 61 million trainable parameters in the model’s entire structure [20].

ResNet50: ResNet is a popular deep-learning model. ResNet has developed a variety of architectures with various layer counts, with 34, 50, 101, 152, and 1202 among them. The most well-known of them, ResNet50, has one fully connected layer at the network’s end and 49 convolution layers. A 224 by 224 image is fed into the input layer. ResNet50 is 96 MB, and has 177 layers in all, including 50 main layers and 25.6 million trainable parameters [21].

GoogleNet: One of the deep learning models, GoogleNet, has 22 layers and aims to have less computational complexity than other CNN architectures. Variable receptive fields, or initial layers, are produced by various core sizes in this methodology. This has taken advantage of the chance to include sparse correlation models in the new feature map stack [22].

EfficientNet-b0: A CNN architecture called EfficientNet was trained using more than 14 million images from the ImageNet database. In contrast to other sophisticated models, the EfficientNet model aims to reduce the model’s size while scaling the depth, width, and resolution to produce more effective results. The EfficientNet group consists of eight models, ranging in complexity from B0 to B7. As the number of models rises, the number of calculated parameters stays relatively flat while accuracy rises noticeably [23].

MobileNetV2: A deep learning model called MobileNetV2 with 53 deep layers and a 13 MB file size is fed by an input layer with 224 × 224 image dimensions. The model’s entire structure consists of 3.5 million trainable parameters [24].

ShuffleNet: The ShuffleNet model is a deep learning model that includes the bottleneck structure. Compared to other CNN architectures, ShuffleNet is more straightforward and has fewer parameters. Additionally, deep convolution applied only in the bottleneck feature map is appropriate for low-power mobile devices [25].

### 2.4. Classifiers

Six different classifiers that are frequently used in the literature were used in the study. These classifiers are briefly explained in turn.

Fine Tree: Decision trees (DT) use the sorting approach for classification, while conventional classifiers use the neural and statistical approaches. Instead of making a single complex decision, simple chains of decisions are made based on the outcomes of sequential tests. The pruning of the tree structure is a crucial step in developing DT. The DT classifier creates a massive and intricate tree structure by segmenting the training data into subsets containing just one class. The error rate for the entire tree will decrease as the error rate in the lower branches decreases. The tree with the lowest error rate is obtained after the pruning process. One of these techniques is the fine-type decision tree [26].

Linear Discriminant (LD): LD is a deep learning architecture for classifying data that looks for a linear combination of variables that best separates classes. Projecting high-dimensional data linearly onto a lower-dimensional space is the underlying idea behind linear dimension-reduction techniques [27].

Naive Bayes (NB): NB is one of the machine learning algorithms for Bayesian text classification that is the simplest, clearest, and most widely applicable. This method can calculate the likelihood that a sample belongs to the class value of the target attribute [28].

Support Vector Machines (SVM): SVM is a popular machine-learning algorithm for classification problems. A supervised learning algorithm, SVM operates without prior distribution knowledge and is based on statistical learning theory. The benefits include a high accuracy rate and no overfitting issues [29].

K-Nearest Neighbor (KNN): The KNN algorithm is one of the most well-known and widely used machine learning algorithms. The similarity between the chosen and closest features is used for classification. The value of k discovered in this instance is expressed with a number, such as three or five [30].

### 2.5. Proposed Model

**Figure 3:**
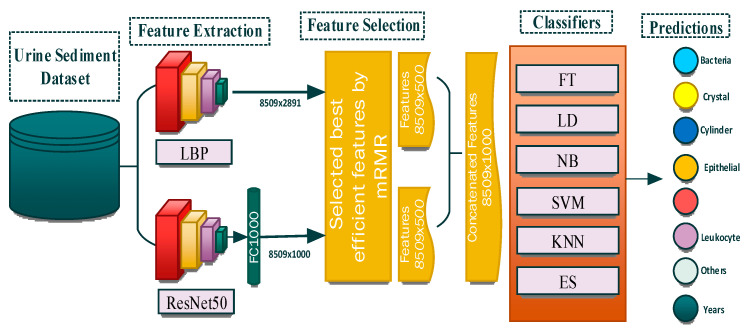
Proposed model.

A tissue and CNN-based hybrid model was developed to classify eight different particles obtained from the urine sediment test. The aim was to extract different features of the images by using two different feature extraction methods. In the proposed model, the texture-based LBP method and CNN-based ResNet50 model were used for the feature extraction. Using the LBP method, 2891 features were obtained from each of the images in the urine sediment data set. Since the number of images in the urine sediment data set is 8509, the size of the feature map obtained in the LBP method is 8509 × 2891. The number of features extracted in the ResNet50 architecture is 1000 in each image. In the ResNet50 architecture, the features are taken from the FC1000 layer. The size of the feature map extracted using the ResNet50 architecture was 8509 × 1000. In order for the proposed model to work faster and produce more effective results, the optimization step was performed before the feature merging process. A total of 500 features were selected from each of the feature maps obtained from the mRMR method, the LBP method, and the ResNet50 architecture. The selected features are then combined. As a result, a feature map of 8509 × 1000 was obtained. Thanks to this joining process, different features extracted by different methods are brought together. The model’s performance is greatly enhanced by this technique. Finally, the optimized and combined feature map was classified in different classifiers accepted in the literature. The flow chart of the proposed model is presented in Figure 3.

The most important factor in using the ResNet50 architecture as the basis for the proposed model is that the highest accuracy value among the pre-trained models used in the study was achieved in this architecture. Using the textural-based LBP approach, feature extraction was also carried out to improve the performance of the suggested model. The features of the same images obtained by ResNet50 and LBP methods were combined. This has increased the performance of the proposed model.

## 3. Results

A tissue and CNN-based hybrid model was developed to classify eight different particles obtained from the urine sediment test. In the developed model, feature extraction was performed using the texture-based LBP method and the CNN-based ResNet50 architecture. While the number of features extracted for each image using the LBP method is 2891, the number of features extracted for each image using the ResNet50 architecture is 1000. In the proposed model, 500 features were selected for each image from each feature map extracted using the mRMR method, LBP method, and ResNet50 architecture, and these features were combined. The optimized and combined feature map was finally classified in six different classifiers. In order to compare the performance of the proposed model, results were obtained in six different pre-trained models. In addition, feature maps were obtained from the last FC layers of six different pre-trained models, and the obtained feature maps were classified in six different classifiers. Finally, the features obtained by the LBP method were similarly classified in six different classifiers.

In the study, different parameters were used to measure the performance of the models. These parameters were Accuracy, Sensitivity, Sensitivity, Specificity, False Discovery Rate (FDR), False Positive Rate (FPR), False Negative Rate (FNR), and F1-score.

### 3.1. Test Results of Pre-Trained Models

**Figure 4:**
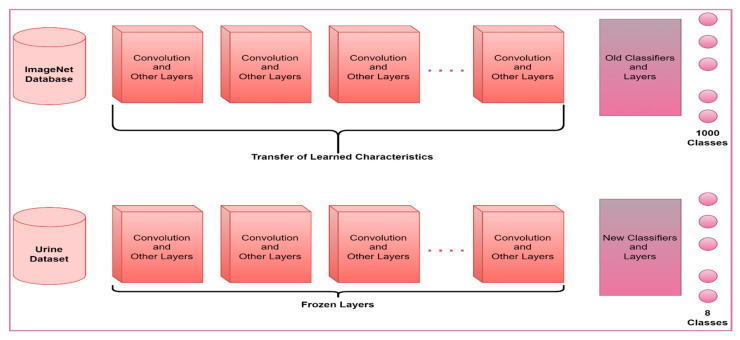
Layer update on pre-trained models.

In this section, six different pre-trained models accepted in the literature were used to classify eight different particles obtained from the urine sediment test. Models used in the study were AlexNet, ResNet50, GoogleNet, ShuffleNet, EfficientNetb0, and MobileNetV2. In these models, ImageNet weights are used instead of randomly choosing initial weights. In this way, the cost and time process of the model trainer from scratch were minimized. The last layers of the architectures used in the study were rearranged according to the eight classes used in the study. The softmax layer, which is frequently used in pre-trained models, is used as the activation function in the updated layers. SGDM optimization, a batch-size size of 16, and a learning rate of 0.001 were chosen. In addition, 80% of the images in the data set were used for training the models and the remaining 20% for testing. Freezing and updating in layers of pre-trained models are shown in outline in Figure 4.

The accuracy values obtained in the pre-trained models in the study are shown in Table 2.

**Figure 5:**
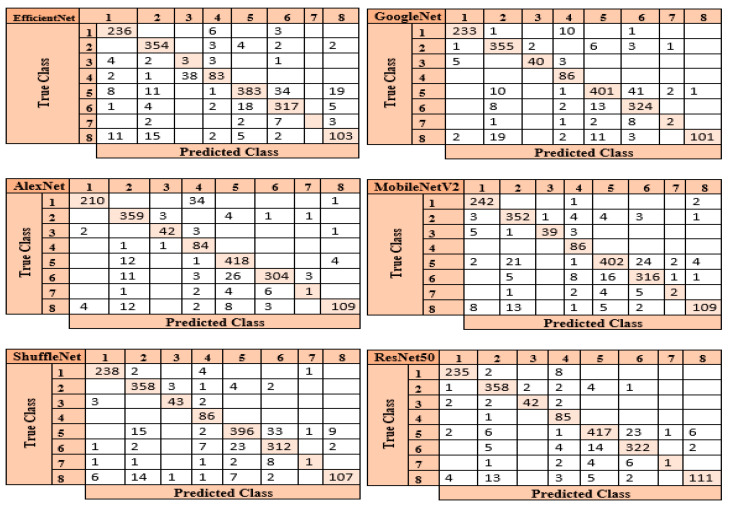
Confusion matrices of the pre-trained models.

In the process of classifying eight different particles obtained from the urine sediment test using pre-trained architectures, the highest accuracy rate was achieved in the ResNet50 model with a value of 92.30%. This accuracy value was followed by MobileNetV2 with 90.95%, GoogleNet with 90.60%, ShuffleNet with 90.54%, AlexNet with 89.72%, and EfficientNetb0 with 88.95%. Confusion matrices obtained in pre-trained models are presented in Figure 5.

The ResNet50 architecture achieved the maximum performance in categorizing test images when the confusion matrices shown in Figure 5 are investigated. The ResNet50 architecture predicted 1571 correctly and 125 incorrectly out of 1696 images allocated for testing. The class where the ResNet50 architecture has the lowest performance is the “Others” (7) class. The reason for the lowest performance in this class is due to the small number of data. The architecture that provides the lowest accuracy among the pre-trained models is EfficientNetb0. Of the 1696 images allocated for this architectural test, EfficientNetb0 predicted 1479 correctly and 217 incorrectly. The class where the EfficientNetb0 architecture has the lowest performance is the “Others” (7) class. This architecture did not correctly predict any images in the “Others” (7) class.

### 3.2. Classification of Feature Maps Extracted Using Pre-Trained Models in Different Classifiers

**Figure 6:**
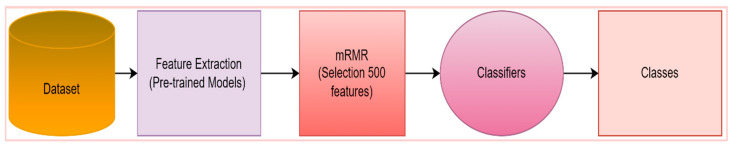
Flowchart of Pre-trained models + mRMR + classifiers.

In the proposed method, ResNet50 architecture, which is one of the CNN-based models, is used for feature extraction. In this section, feature extraction was performed with six different pre-trained models used in the study. Using pre-trained models, 1000 features were obtained for each image from the data set with 8 different particles obtained from the urine sediment test. The last fully connected layers of the pre-trained models were used for feature extraction. This layer is the layer before the classification layer in pre-trained architectures. A total of 500 of the features from pre-trained architectures were selected by the mRMR method. In this way, since unnecessary features are eliminated, the model will run faster, and more effective results will be obtained. As a result, after feature extraction with pre-trained models and optimization process with the mRMR method, the size of the existing feature map became 8509 × 500. Finally, the 8509 × 500 feature map was classified by different classifiers. The relevant process is summarized in Figure 6.

The accuracy values in Table 3 were obtained after the feature map, which was extracted with pre-trained models and optimized with the mRMR, was classified by the six different classifiers used in the study.

**Figure 7:**
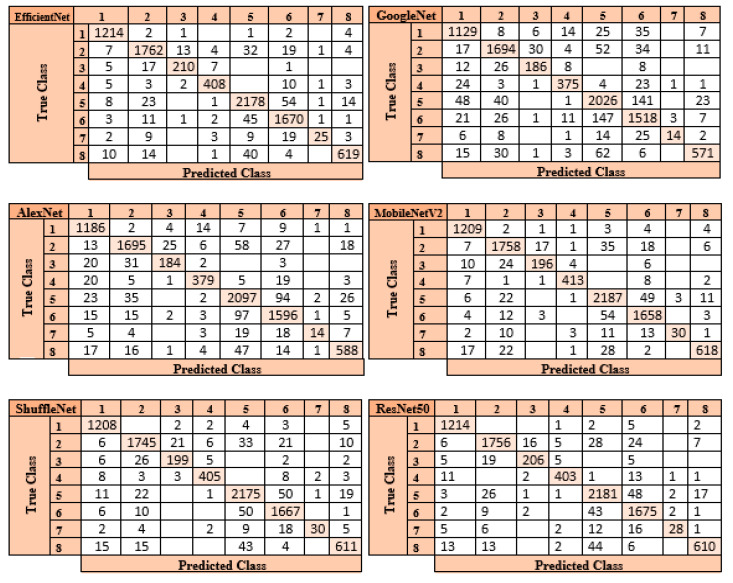
Confusion matrices of the Pre-trained Models+ SVM + Classifiers.

Examining the accuracy values in Table 3 reveals that the SVM classifier achieves the highest accuracy rate after the features from six different pre-trained models are optimized using the mRMR method. Figure 7 displays the confusion matrices in each architecture’s SVM classifier.

The ResNet50 architecture is the one that performs the best when the confusion matrices shown in Figure 7 are studied. In the confusion matrix created by the SVM classifier after the feature map created by the ResNet50 architecture was optimized using the mRMR approach, the SVM classifier correctly identified 8073 out of 8509 images and incorrectly identified 436.

### 3.3. Classification of Feature Maps Extracted Using Pre-Trained Models in Different Classifiers

**Figure 8:**
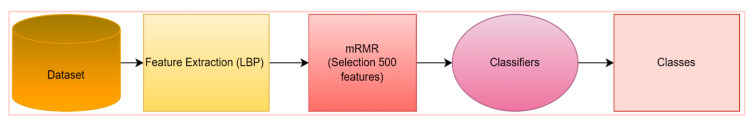
LBP—mRMR Flowchart.

Another method used for feature extraction in the proposed method is the texture-based LBP method. Using the LBP method, 2891 features were obtained for each image from the data set with 8 different particles obtained from the urine sediment test. A total of 500 of these features were selected by the mRMR method. In this way, since unnecessary features are eliminated, the model will run faster, and more effective results will be obtained. As a result, after feature extraction with the LBP method and the optimization process with the mRMR method, the size of the existing feature map became 8509 × 500. The relevant process is summarized in Figure 8.

The accuracy values in Table 4 were obtained after the feature map extracted by the LBP method and optimized by mRMR were classified by the six different classifiers used in the study.

**Figure 9:**
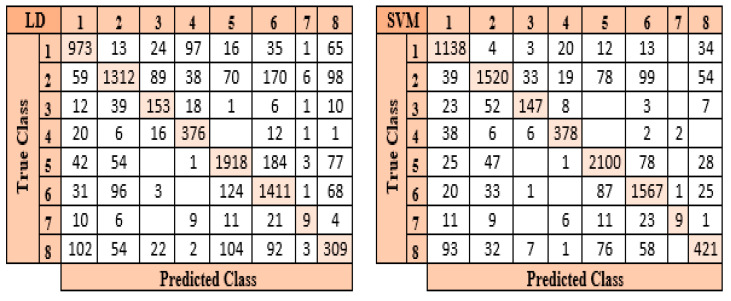
Confusion matrices of the classifiers with the highest accuracy in the LBP method.

After feature extraction using the LBP method and the optimized feature map with the mRMR method, the optimized feature map with a size of 8509 × 500 was classified by the six different classifiers used in the study. In the classification process of eight different particles obtained from the urine sediment test, the highest accuracy value was reached in the SVM classifier at 85.6%. This accuracy value was followed by LD at 75.9%, KNN at 75.0%, ES NB at 74.2%, and FT at 61.8%, respectively. Confusion matrices belonging to SVM and LD classes, where the highest accuracy values are obtained, are presented in Figure 9.

When the confusion matrices presented in Figure 9 are examined, it can be seen that the SVM classifier estimated 7280 of 8509 images correctly and 1229 of them incorrectly. When classifying the features retrieved by the LBP method, it was found that the SVM classifier performs better than other classifiers. LD is the most successful classifier after the SVM classifier in classifying the features extracted by the LBP method. The LD classifier predicted 6461 of 8509 images correctly and 2048 incorrectly. The LD classifier was the second-highest accuracy classifier.

### 3.4. The Results Obtained in the Proposed Model

**Figure 10:**
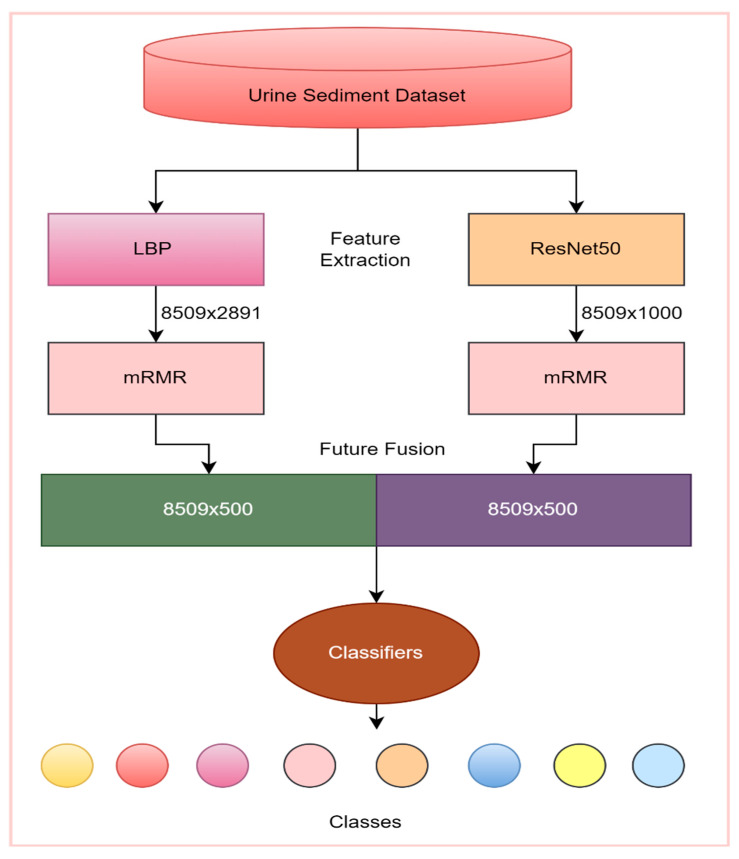
Flow chart of the proposed model.

In the proposed method, the texture-based LBP method and ResNet50 architecture are used for feature extraction. While the number of features extracted for each image with the LBP method is 2891, the number of features taken from the FC1000 layer of the ResNet50 architecture is 1000. In order for the proposed method to work faster and more effectively, 500 features were selected from each method by the mRMR method. Then, these selected features were combined and classified by different classifiers. As a result, since there are 8509 images in the data set, the size of the obtained feature map is 8509 × 1000. The relevant process is summarized in Figure 10.

The accuracy values in Table 5 were obtained after the feature map obtained using the proposed method was classified at different classifiers.

**Figure 11:**
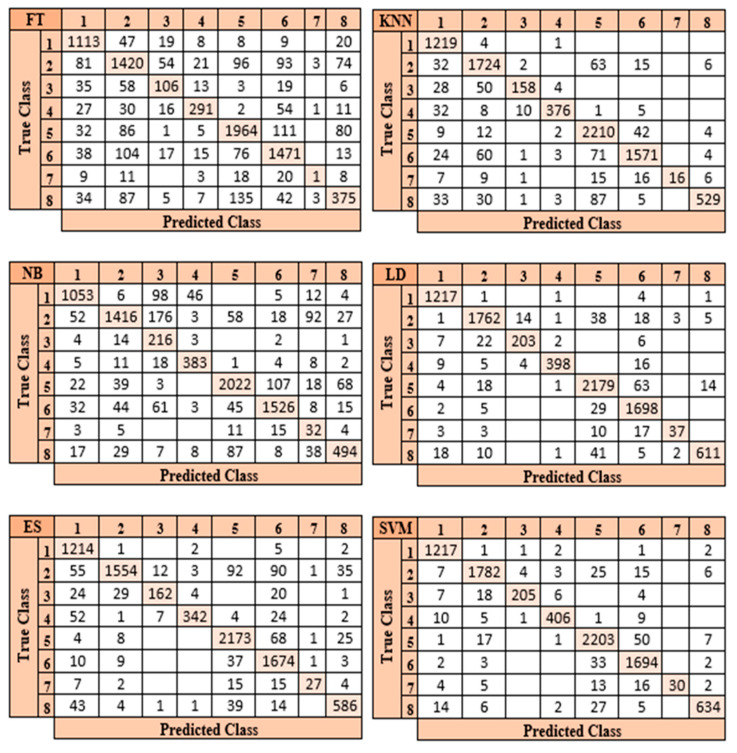
Confusion matrices of the proposed model.

In the developed model, the highest accuracy rate of 96% was achieved in the SVM classifier in the classification process of eight different particles obtained from the urine sediment test. This accuracy value was followed by LD at 95.3%, KNN at 91.7%, ES at 90.9%, NB at 83.9%, and FT at 79.2%, respectively. Confusion matrices obtained by six different classifiers in the proposed model are presented in Figure 11.

When the confusion matrices shown in Figure 11 are analyzed, it becomes clear that the SVM classifier in the suggested model is the most effective classifier. While the SVM classifier classified 8441 of 8509 images correctly, it classified 68 images incorrectly. The proposed method correctly predicted 1217 of the 1224 Bacteria (1) images in the SVM classifier and predicted 7 of them incorrectly. A total of 1782 of 1842 images in the Crystal (2) class were predicted correctly and 60 of them were incorrectly predicted. Of the 240 images in the Cylinder (3) class, 205 were predicted correctly and 35 were predicted incorrectly. Of the 432 images in the Epithelial (4) class, 406 were predicted correctly and 26 were predicted incorrectly. Of the 2279 images in the Erythrocyte (5) class, 2203 were predicted correctly and 76 were predicted incorrectly. Of the 1734 images in the Leukocyte (6) class, 1694 were predicted correctly and 40 were predicted incorrectly. Of the 70 images in the “Others” (7) class, 30 were guessed correctly and 40 were guessed incorrectly. Of the 688 images in the Yeast (8) class, 634 were predicted correctly and 54 were predicted incorrectly. The class in which the proposed model failed the most was the “Others” class. The reason for obtaining the lowest performance rate in this class is due to the small number of data in this class. The performance measurement metrics obtained in the SVM classifier in the developed model are presented in Table 6.

When the performance metrics of the proposed model are evaluated, it is observed that the highest accuracy value is 99.42% in the Bacteria class and the lowest accuracy value is 42.85% in the “Others” class.

## 4. Discussion

Urine sediment analysis is considered very important by experts for the diagnosis and follow-up of many diseases. This analysis contains important information about kidney diseases such as kidney stones, kidney failure, and prostate diseases. In urinalysis, the shape, amount, or number of particles in the urine is critical for specialists to diagnose the disease, at what stage it is, and to determine the method to be used for treatment [31,32].

**Figure 12:**
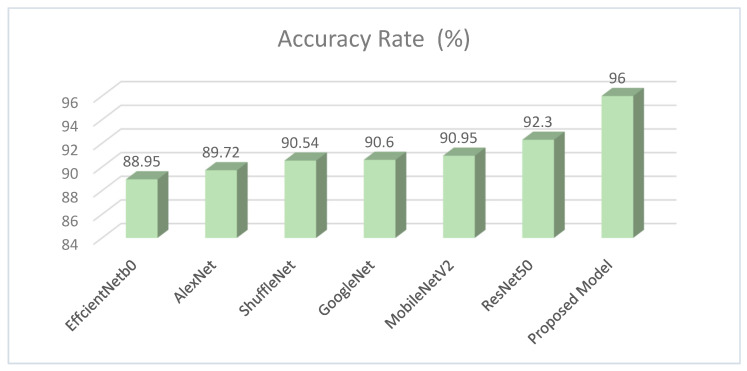
Accuracy rates of models.

The examination and classification of biomedical images with deep learning techniques have been quite common in recent years. The reason for this is the ability of deep learning techniques to classify with a higher accuracy than traditional methods. In this study, urine sediment particle images were classified for the first time with a hybrid method of deep learning and tissue-based, proposed in this study. The results obtained in the study with the pre-trained models and the proposed model are compared in Figure 12.

The proposed model is also compared with other studies on the classification of urine analysis images in Table 7.

As can be seen from Table 7, the highest performance in the literature was not obtained with the method we suggested. However, the number of images in the data set used in our study is more than 10 times the number of images in the study with the highest accuracy rate in Table 7, and the number of classes is exactly 2 times. Considering all these values, we evaluate that the proposed hybrid method can be used for the classification of urine sediment particle images. The biggest factor in achieving a high accuracy value in the proposed model is the combination of CNN and textural-based features. In addition, unnecessary features have been eliminated with the mRMR method to make the proposed model work faster.

Our study does have certain limitations. The most significant limitation we see is that the data set we used in the experiments was created with urine samples obtained from a microbiology laboratory in a single hospital. Another limitation is the uneven distribution of the number of images within each class in the data set.

A data set in which the number of images in each class in the data set is distributed properly and the data are created in a multicenter format is among our future studies.

## 5. Conclusions

The human population is increasing day by day all over the world. Therefore, the workload of experts is increasing and there are is a scarcity of experts everywhere. Therefore, it is of great importance to bring computer-aided systems to the fore, especially in the biomedical field. Thanks to these computer-aided systems, the workload of the experts will be lightened, and the pre-diagnosis process will be shortened. The shortening of the diagnosis time will allow for the early initiation of treatment. Therefore, in this study, a computer-aided hybrid model was developed. The proposed model consists of the steps of feature extraction with textural LBP method and CNN-based ResNet50, combining these extracted features, feature selection from the combined feature map with mRMR method, and finally classification of the optimized feature map in the SVM. The developed model was compared with similar studies in the literature and with pre-trained models. A high accuracy value of 96% was obtained in classifying the particles in the proposed model urine-sediment test. The most important factor in achieving a high accuracy value for our proposed CNN and textural-based hybrid model is the extraction of different features with two different methods and then combining them. In previous studies, either CNN-based methods or textural-based methods were used separately. In addition, unnecessary features in the feature map were eliminated by using the mRMR method in the size reduction step in order to make our proposed model work faster and more effectively. Measuring the performance of the feature map on different classifiers was another factor that increased the impact of our model. Considering the eight class urinary-sediment data set used in the study, it shows that the accuracy value obtained in the proposed model can be used to classify the particles in the urinary sediment test.

## Figures and Tables

**Table 1:** Urine sediment particle classes and numbers in the data set.

Bacteria	Crystal	Cylinder	Epithelial	Erythrocyte	Leukocyte	Yeast	Others	Total
1224	1842	240	432	2279	1734	688	70	8509

**Table 2:** Accuracy values of pre-trained models (%).

EfficientNetb0	AlexNet	ShuffleNet	GoogleNet	MobileNetV2	ResNet50
88.95	89.72	90.54	90.60	90.95	92.30

**Table 3:** Accuracy of Pre-trained Models+ SVM + Classifiers (%).

	FT	LD	NB	SVM	KNN	ES
EfficientNetb0	78.8	94.0	82.3	95.0	92.5	91.7
AlexNet	73.9	88.1	64.3	91.0	84.5	84.1
ShuffleNet	79.7	92.6	75.5	94.5	90.9	90.3
GoogleNet	72.8	85.6	66.4	88.3	83.5	81.2
MobileNetV2	76.6	92.9	83.0	94.8	92.7	92.0
ResNet50	79	94.5	80.1	94.9	91.3	91.1

**Table 4:** Accuracy rate of LBP + mRMR (%).

	FT	LD	NB	SVM	KNN	ES
LBP + mRMR	61.8	75.9	72.4	85.6	75.0	74.2

**Table 5:** Accuracy rate of LBP-mRMR + ResNet50-mRMR (%).

	FT	LD	NB	SVM	KNN	ES
Proposed Model	79.2	95.3	83.9	96	91.7	90.9

**Table 6:** Performance metrics of the proposed method (%).

	Accuracy	Sensitivity	Specificity	FPR	FDR	FNR	F1
Bacteria	99.42	96.43	99.90	0.09	0.57	3.56	97.90
Crystal	96.74	97.00	99.10	0.89	3.25	2.99	96.87
Cylinder	85.41	97.15	99.57	0.42	14.58	2.84	90.90
Epithelial	93.98	96.66	99.67	0.32	6.01	3.33	95.30
Erythrocyte	96.66	95.69	98.77	1.22	3.33	4.30	96.17
Leukocyte	97.69	94.42	99.40	0.59	2.30	5.57	96.03
Others	42.85	100	99.52	0.47	57.14	0	60
Yeast	92.15	97.09	99.31	0.68	7.84	2.90	94.55

**Table 7:** Studies in the literature on the classification of urine particle images.

Reference	Method	Number of Images	Number of Class	Accuracy (%)
Li et al. [6]	RetinaNet	15,360	16	88.65
Liang et al. [7]	Faster R-CNN, SSD	5376	7	84.10
Nagai et al. [8]	Xception	441	15	84.40
Khalid et al. [10]	DenseNet, InceptionV3	820	4	96.50
Lee et al. [11]	DenseNet	263	3	90.90
Ji et al. [12]	US-RepNet	429,605	16	94.00
Li et al. [13]	LeNet-5	2551	4	92.00
Proposed Model	Resnet50 + LBP + mRMR + SVM	8509	8	96.00

## Data Availability

Not applicable.
